# Risk of Severe Acute Kidney Injury According to the Presence of Nephrotic-Range Proteinuria in Patients with Liver Cirrhosis: A Retrospective Cohort Study (2016–2025)

**DOI:** 10.3390/medicina62040797

**Published:** 2026-04-21

**Authors:** Seong Gyu Kim, Sang Gyu Kwak

**Affiliations:** 1 Division of Nephrology, Department of Internal Medicine, Daegu Catholic University School of Medicine, Daegu 42472, Republic of Korea; ksgnephro@cu.ac.kr; 2 Department of Medical Statistics, Daegu Catholic University School of Medicine, Duryugongwon-Ro 17-Gil 33, Nam-Gu, Daegu 42472, Republic of Korea

**Keywords:** acute kidney injury, cirrhosis, chronic kidney disease, Child–Pugh class, KDIGO, nephrotic-range proteinuria, urine protein-to-creatinine ratio

## Abstract

*Background and Objectives:* Acute kidney injury (AKI) is a frequent and life-threatening complication in patients with liver cirrhosis (LC). Nephrotic-range proteinuria may reflect underlying structural renal vulnerability; however, its association with AKI severity in cirrhosis remains unclear. *Materials and Methods:* We conducted a retrospective cohort study of 408 adults with LC admitted to a tertiary referral hospital between January 2016 and December 2025. Nephrotic-range proteinuria was defined as a urine protein-to-creatinine ratio (UPCR) ≥3.5 g/g measured within 7 days before or at admission. AKI was staged using serum creatinine-based Kidney Disease: Improving Global Outcomes criteria. Baseline creatinine was defined as the lowest value within 7 days before admission; if unavailable, the lowest stable value within the preceding 3 months was used. Severe AKI was defined as KDIGO stage 2–3. Multivariable logistic regression was performed to evaluate the association between nephrotic-range proteinuria and severe AKI after adjustment for age, sex, diabetes mellitus, hypertension, chronic kidney disease (CKD), sepsis, ICU admission, and Child–Pugh class. *Results:* Of the 408 patients, 56 (13.7%) had nephrotic-range proteinuria. Severe AKI occurred more frequently in patients with nephrotic-range proteinuria than in those without (39.3% vs. 21.9%), corresponding to an absolute risk difference of 17.4 percentage points (*p* = 0.008). In the adjusted model, nephrotic-range proteinuria was associated with a higher likelihood of severe AKI (adjusted odds ratio [OR], 2.27; 95% confidence interval [CI], 1.17–4.41; *p* = 0.015). CKD (adjusted OR, 2.26; 95% CI, 1.33–3.81; *p* = 0.002), ICU admission (adjusted OR, 2.03; 95% CI, 1.22–3.39; *p* = 0.007), and Child–Pugh class C versus A (adjusted OR, 3.50; 95% CI, 1.37–8.93; *p* = 0.009) were also significantly associated with severe AKI. *Conclusions:* Among hospitalized patients with LC, nephrotic-range proteinuria was associated with a higher likelihood of severe AKI. Quantitative proteinuria assessment may help identify patients at increased risk of advanced renal dysfunction, although causal inference is limited by the retrospective observational design.

## 1. Introduction

Liver cirrhosis (LC) represents the final stage of chronic liver disease and is characterized by progressive hepatic fibrosis, portal hypertension, and multi-organ dysfunction. Among its extrahepatic complications, acute kidney injury (AKI) is one of the most frequent and clinically devastating events. The reported incidence of AKI in hospitalized patients with cirrhosis ranges from approximately 20% to over 50%, depending on disease severity and diagnostic criteria, and is consistently associated with increased short-term mortality and healthcare burden [1,2,3]. Importantly, not only the occurrence but also the severity of AKI has emerged as a major determinant of prognosis, with higher AKI stages conferring substantially worse clinical outcomes [2,4].

The pathophysiology of AKI in cirrhotic patients is complex and involves hemodynamic, inflammatory, and structural mechanisms. Systemic vasodilation and splanchnic arterial pooling reduce effective arterial blood volume and impair renal perfusion, while systemic inflammation and bacterial translocation further disrupt renal autoregulation [5,6,7]. In advanced stages, hepatorenal syndrome (HRS) represents a severe functional form of renal failure driven primarily by circulatory dysfunction [6,8]. However, contemporary evidence suggests that AKI in cirrhosis is heterogeneous and may include structural renal injury beyond purely functional impairment [9]. This heterogeneity may partly explain why some patients progress to severe AKI despite similar degrees of liver dysfunction. Traditionally, AKI in cirrhosis has been conceptualized as a predominantly functional disorder driven by circulatory dysfunction, such as splanchnic vasodilation and reduced effective arterial blood volume. However, recent evidence suggests that AKI in cirrhosis is a heterogeneous condition that may also involve structural kidney injury, systemic inflammation, and microcirculatory dysfunction. This evolving paradigm highlights that renal dysfunction in cirrhosis cannot be fully explained by functional mechanisms alone.

Chronic kidney disease (CKD) and AKI are increasingly recognized as interconnected syndromes, with pre-existing renal impairment amplifying susceptibility to acute renal insults and worsening outcomes [10]. Reduced nephron reserve in patients with underlying kidney damage may predispose them to more severe forms of AKI when exposed to hemodynamic instability, infection, or nephrotoxic agents.

Nephrotic-range proteinuria, a marker of substantial glomerular injury, may be accompanied by hypoalbuminemia and reduced renal reserve. Beyond serving as a marker of kidney damage, proteinuria may directly promote tubular inflammation and oxidative stress, thereby accelerating renal functional decline [11]. In non-cirrhotic populations, nephrotic-range proteinuria has been associated with an increased risk of AKI and adverse renal outcomes [12,13]. In cirrhotic patients, significant proteinuria may indicate underlying structural renal vulnerability in addition to the well-recognized circulatory disturbances of advanced liver disease. Hypoalbuminemia may further exacerbate effective arterial underfilling, while structural glomerular injury may reduce renal reserve capacity, potentially predisposing patients to severe AKI during acute stress.

Despite these plausible biological mechanisms, the impact of nephrotic-range proteinuria on AKI severity in patients with cirrhosis remains insufficiently studied. Most previous investigations have focused primarily on the overall incidence of AKI rather than the development of severe AKI (KDIGO stage 2–3), which carries the greatest prognostic significance [1,2]. Importantly, severe AKI is more strongly associated with adverse clinical outcomes, including increased mortality and prolonged hospitalization. Therefore, focusing specifically on severe AKI may provide more clinically meaningful insights into high-risk patient subgroups. In this context, our study specifically evaluates the association between nephrotic-range proteinuria and severe AKI, addressing a clinically important yet underexplored aspect of cirrhosis-related kidney injury. Furthermore, it remains unclear whether nephrotic-range proteinuria is associated with severe AKI after adjustment for established risk factors such as CKD, systemic infection, and advanced liver dysfunction.

Therefore, the present study aimed to evaluate whether the presence of nephrotic-range proteinuria is associated with the development of severe AKI (KDIGO stage 2–3) in hospitalized patients with LC. We hypothesized that significant proteinuria identifies a high-risk subgroup characterized by increased structural renal vulnerability and a greater likelihood of advanced AKI.

## 2. Materials and Methods

### 2.1. Study Design and Setting

This retrospective cohort study was conducted at Daegu Catholic University Hospital, a tertiary referral academic medical center in the Republic of Korea. We reviewed electronic medical records (EMR) and the institutional clinical data warehouse (CDW) to identify eligible patients admitted between 1 January 2016 and 31 December 2025. The study was designed to evaluate the association between nephrotic-range proteinuria and the development of severe AKI in patients with LC.

The study protocol was approved by the Institutional Review Board of Daegu Catholic University Medical Center (DCUMC 2026-03-014). Informed consent was waived due to the retrospective analysis of de-identified data.

### 2.2. Study Population

Adult patients aged 18 years or older who were diagnosed with LC and admitted to Daegu Catholic University Hospital between 1 January 2016 and 31 December 2025 were screened for eligibility. LC was identified based on a combination of International Classification of Diseases, 10th Revision (ICD-10) diagnostic codes and supporting clinical evidence, including radiologic findings consistent with cirrhosis or documentation by a hepatology specialist in the EMR. To avoid duplication and clustering effects, only the first hospitalization during the study period was considered for patients with multiple admissions.

Patients were included if quantitative urine protein measurements were available prior to or at the time of the index admission, allowing for the assessment of nephrotic-range proteinuria status. Individuals receiving maintenance hemodialysis or peritoneal dialysis before admission were excluded to avoid confounding by pre-existing end-stage renal disease. Patients without sufficient baseline serum creatinine data required to determine AKI status were also excluded. Additionally, cases with missing essential variables necessary for defining nephrotic-range proteinuria or staging AKI were excluded from the final analysis. Patients who had undergone liver transplantation prior to admission were not included.

After applying these inclusion and exclusion criteria, a total of 408 patients were included in the final analysis. The patient selection process and reasons for exclusion are presented in Figure S1 (Supplementary Material).

We performed a complete-case analysis. Patients were excluded if data required to define the exposure (UPCR), baseline kidney function, peak serum creatinine for KDIGO staging, or covariates included in the multivariable model were missing. The number of excluded patients and reasons for exclusion are summarized in a study flow diagram (Figure S1).

### 2.3. Definition of Nephrotic-Range Proteinuria

Nephrotic-range proteinuria was defined as a urine protein-to-creatinine ratio (UPCR) ≥ 3.5 g/g, consistent with the conventional diagnostic threshold for nephrotic-range proteinuria. UPCR measurements were obtained from spot urine samples recorded in the EMR. When multiple UPCR values were available within seven days prior to admission, the value closest to the index admission date was used to reflect the patient’s clinical status at presentation.

Because this study was conducted retrospectively using routinely collected clinical data, serum lipid levels and 24-h urine protein quantification were not uniformly available for all patients. Therefore, patients were classified according to the presence of nephrotic-range proteinuria rather than the full clinical diagnostic criteria for nephrotic syndrome. This approach allowed for consistent classification across the study population while minimizing selection bias due to missing laboratory parameters.

Patients were subsequently categorized into groups with and without nephrotic-range proteinuria according to this predefined threshold.

### 2.4. Baseline Kidney Function and AKI Definition

Baseline kidney function was defined as the lowest serum creatinine value measured within 7 days prior to hospital admission. If no serum creatinine measurement was available within this 7-day window, the lowest stable value documented within the preceding 3 months was used as the reference baseline. This hierarchical approach was selected to approximate stable pre-admission kidney function while minimizing misclassification of pre-existing renal dysfunction. Patients without any available creatinine measurement within these predefined periods were excluded from the analysis.

AKI was defined and staged according to the Kidney Disease: Improving Global Outcomes (KDIGO) clinical practice guideline [14] and applied in patients with cirrhosis in accordance with the recommendations of the International Club of Ascites [8]. AKI staging was based on changes in serum creatinine levels.

Urine output criteria were not incorporated because accurate hourly urine output data were not consistently available in the retrospective medical records. The use of serum creatinine–based criteria alone has been widely adopted in retrospective cirrhosis cohorts in which urine output documentation is incomplete [8,14].

AKI staging was determined using the peak serum creatinine level recorded during hospitalization relative to the defined baseline value. Stage 1 was defined as a 1.5–1.9-fold increase from baseline or an absolute increase of ≥0.3 mg/dL. Stage 2 was defined as a 2.0–2.9-fold increase from baseline. Stage 3 was defined as a ≥3.0-fold increase from baseline, a serum creatinine level ≥ 4.0 mg/dL, or initiation of renal replacement therapy during hospitalization [14].

Severe AKI was predefined as KDIGO stage 2 or stage 3. This categorization was selected based on prior evidence demonstrating significantly worse clinical outcomes in patients with stage 2–3 AKI compared with those with stage 0–1 in cirrhotic populations [1,2].

### 2.5. Covariates and Clinical Variables

Baseline demographic and clinical variables were extracted from the EMR to account for potential confounding factors associated with the development of severe AKI in patients with LC.

Demographic variables included age and sex. Age was treated as a continuous variable, and sex was categorized as male or female. Comorbid conditions known to influence renal outcomes were recorded, including diabetes mellitus, hypertension, and CKD. CKD was defined based on either (1) a documented clinical diagnosis of CKD in the EMR prior to admission or (2) a preadmission estimated glomerular filtration rate (eGFR) < 60 mL/min/1.73 m^2^ calculated from the most recent stable serum creatinine measurement available within 3 months before admission. When multiple preadmission creatinine values were available, the value used to define baseline kidney function was applied to estimate eGFR. These variables were included because pre-existing renal dysfunction and metabolic comorbidities are established risk factors for AKI progression.

Clinical severity variables reflecting acute physiological stress were also collected. Sepsis was defined based on a physician-documented diagnosis of systemic infection requiring treatment during hospitalization, supported by clinical evidence such as administration of intravenous antibiotics and/or positive microbiological culture results. Admission to the intensive care unit (ICU) was recorded as a surrogate marker of critical illness severity.

Liver disease severity was assessed using laboratory parameters and clinical findings. Total bilirubin, serum albumin, and international normalized ratio (INR) were collected at admission. The Child–Pugh classification was calculated based on standard criteria, incorporating bilirubin, albumin, INR, ascites, and hepatic encephalopathy when documented. Child–Pugh class was entered into regression models as a categorical variable (A, B, C), with class A serving as the reference group. This categorization allowed assessment of whether advanced hepatic dysfunction independently influenced the risk of severe AKI.

Ascites and a history of variceal bleeding were recorded as indicators of portal hypertension and decompensated cirrhosis. These factors were included to account for the hemodynamic and systemic consequences of advanced liver disease that may predispose patients to renal hypoperfusion.

All covariates included in the multivariable model were selected a priori based on clinical relevance and prior literature demonstrating their association with AKI or adverse outcomes in cirrhotic populations.

### 2.6. Outcome Measures

The primary outcome of this study was the occurrence of severe AKI, defined as KDIGO stage 2 or stage 3 during hospitalization. Severe AKI was selected as the primary endpoint because previous studies have demonstrated that higher AKI stages are more strongly associated with adverse clinical outcomes, including increased mortality and prolonged hospitalization, compared with mild AKI. The staging of AKI was determined based on the peak serum creatinine level during hospitalization relative to the predefined baseline value.

Secondary outcomes included the overall occurrence of AKI (any KDIGO stage ≥ 1) and the distribution of AKI stages (stage 0–3) according to the presence or absence of nephrotic-range proteinuria. The distribution of AKI stages was analyzed to explore whether nephrotic-range proteinuria was associated not only with the occurrence of AKI but also with a shift toward more severe stages of renal dysfunction. All outcomes were assessed during the index hospitalization.

### 2.7. Statistical Analysis

Continuous variables were assessed for normality using the Shapiro–Wilk test. Normally distributed variables are presented as mean ± standard deviation, whereas non-normally distributed variables are presented as median with interquartile range. Categorical variables are presented as number (percentage).

Baseline characteristics were compared between patients with and without nephrotic-range proteinuria. Continuous variables were analyzed using Student’s *t*-test or Welch’s *t*-test for normally distributed data, and the Mann–Whitney U test for non-normally distributed data. Categorical variables were compared using the chi-square test or Fisher’s exact test, as appropriate. To quantify the magnitude of baseline differences between groups independent of sample size, standardized mean differences (SMDs) were calculated. An SMD < 0.1 was considered negligible, 0.1–0.2 small, 0.2–0.5 moderate, and >0.5 large imbalance. SMDs were used descriptively to assess baseline imbalance, although no propensity score–based adjustment was performed.

The primary analysis evaluated the association between nephrotic-range proteinuria and severe AKI (KDIGO stage 2–3). Crude odds ratios (ORs) with 95% confidence intervals (CIs) were first calculated using univariable logistic regression.

Subsequently, multivariable logistic regression analysis was performed to determine whether nephrotic-range proteinuria remained significantly associated with severe AKI after adjustment for clinically relevant covariates. Covariates included age, sex, diabetes mellitus, hypertension, CKD, sepsis, ICU admission, and Child–Pugh class. Because serum albumin is incorporated into the Child–Pugh classification, it was not included separately in the multivariable model to avoid redundancy. Child–Pugh class was entered as categorical dummy variables, with class A serving as the reference category. Adjusted ORs with 95% CIs were reported. Model calibration was assessed using the Hosmer–Lemeshow goodness-of-fit test. Discrimination was evaluated using the C-statistic (area under the receiver operating characteristic curve). Multicollinearity among covariates was assessed using variance inflation factors (VIFs), with a VIF > 5 considered indicative of substantial multicollinearity.

In secondary analyses, the distribution of AKI stages (0–3) according to nephrotic-range proteinuria status was compared using the chi-square test.

Statistical analyses were primarily performed using R (version 4.5.1; R Foundation for Statistical Computing, Vienna, Austria). Additional model diagnostics (C-statistic and variance inflation factors) were computed using Python (version 3.13.14) (statsmodels and scikit-learn). A two-sided *p*-value < 0.05 was considered statistically significant.

## 3. Results

### 3.1. Baseline Characteristics

Between January 2016 and December 2025, a total of 408 patients with LC met the eligibility criteria and were included in the final analysis. Among them, 56 patients (13.7%) were classified as having nephrotic-range proteinuria (UPCR ≥ 3.5 g/g), whereas 352 patients (86.3%) did not have nephrotic-range proteinuria. Baseline characteristics according to nephrotic-range proteinuria status are presented in Table 1.

The mean age of the study population was similar between groups (64.48 ± 10.22 years in patients without nephrotic-range proteinuria vs. 65.36 ± 10.99 years in those with nephrotic-range proteinuria, *p* = 0.576). The proportion of male patients was also comparable (67.0% vs. 75.0%, *p* = 0.302). The prevalence of diabetes mellitus (38.6% vs. 41.1%, *p* = 0.842) and hypertension (45.5% vs. 46.4%, *p* = 1.000) did not differ significantly between groups. However, CKD was significantly more common in patients with nephrotic-range proteinuria (55.4% vs. 30.7%, *p* < 0.001), with a moderate SMD (0.50), indicating clinically meaningful imbalance.

With respect to liver-related parameters, total bilirubin and INR were comparable between groups. However, serum albumin levels were significantly lower in those with nephrotic-range proteinuria (2.79 ± 0.54 g/dL vs. 3.02 ± 0.50 g/dL, *p* = 0.004; SMD = −0.44). The distribution of Child–Pugh classes did not significantly differ (*p* = 0.458), although a numerically higher proportion of class C patients was observed in those with nephrotic-range proteinuria (25.0% vs. 20.2%).

Baseline renal function differed between groups. Patients with nephrotic-range proteinuria had higher baseline creatinine levels (1.20 ± 0.39 mg/dL vs. 1.03 ± 0.34 mg/dL, *p* = 0.003; SMD = 0.45). As expected, UPCR values were markedly elevated in those with nephrotic-range proteinuria (5.10 ± 1.35 g/g vs. 1.62 ± 0.89 g/g, *p* < 0.001).

### 3.2. AKI Occurrence and Severity

The distribution of AKI stages according to nephrotic-range proteinuria status is shown in Table 2. Overall, 77 patients (21.9%) without nephrotic-range proteinuria and 22 patients (39.3%) with nephrotic-range proteinuria developed severe AKI (KDIGO stage 2–3), corresponding to an absolute risk difference of 17.4 percentage points (*p* = 0.008). When analyzed by individual AKI stages, stage 3 occurred in 10 patients (17.9%) with nephrotic-range proteinuria compared with 24 patients (6.8%) without nephrotic-range proteinuria. The overall distribution of AKI stages differed significantly between groups (*p* = 0.004), suggesting a shift toward more severe renal injury in patients with nephrotic-range proteinuria. The crude OR for severe AKI patients with nephrotic-range proteinuria was 2.31 (*p* = 0.0079), indicating more than twofold higher odds of severe AKI compared with patients without nephrotic-range proteinuria.

### 3.3. Multivariable Logistic Regression Analysis

**Figure 1:**
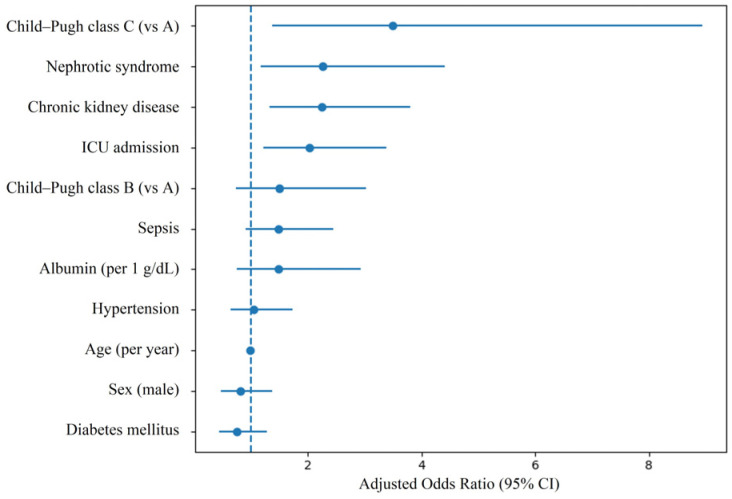
Forest plot of adjusted odds ratios for severe acute kidney injury. Child–Pugh class A was used as the reference category. CI, confidence interval; ICU, intensive care unit.

Multivariable logistic regression analysis for severe AKI is presented in Table 3 and Figure 1. After adjustment for age, sex, diabetes mellitus, hypertension, CKD, sepsis, ICU admission, and Child–Pugh class, nephrotic-range proteinuria remained significantly associated with severe AKI (adjusted OR 2.27, 95% CI 1.17–4.41, *p* = 0.015). CKD was also associated with severe AKI (adjusted OR 2.26, 95% CI 1.33–3.81, *p* = 0.002), as was ICU admission (adjusted OR 2.03, 95% CI 1.22–3.39, *p* = 0.007). Compared with Child–Pugh class A, Child–Pugh class C was significantly associated with severe AKI (adjusted OR 3.50, 95% CI 1.37–8.93, *p* = 0.009), whereas class B was not statistically significant.

Age, sex, diabetes mellitus, hypertension, and sepsis were not significantly associated with severe AKI in the adjusted model. The multivariable model demonstrated acceptable calibration (Hosmer–Lemeshow test, *p* = 0.627) and good discrimination (C-statistic = 0.706). No evidence of significant multicollinearity was observed, with a maximum VIF of 2.557 among included covariates.

## 4. Discussion

In this retrospective cohort study of 408 hospitalized patients with LC, we found that nephrotic-range proteinuria was significantly associated with severe AKI, defined as KDIGO stage 2–3. Severe AKI occurred in 39.3% of patients with nephrotic-range proteinuria compared with 21.9% in those without, corresponding to an absolute risk difference of 17.4 percentage points. After adjustment for demographic characteristics, comorbidities, liver disease severity, and markers of critical illness, nephrotic-range proteinuria remained significantly associated with severe AKI (adjusted OR 2.27, 95% CI 1.17–4.41; *p* = 0.015). Because the outcome was relatively common in this study population, odds ratios may overestimate the magnitude of association compared with risk-based measures. Therefore, the effect size should be interpreted with caution, particularly when translating these findings into clinical risk assessment. Complementary approaches such as risk ratios or marginal effects may provide more intuitive estimates and additional clinical insight in future studies. These findings suggest that substantial proteinuria may identify a subgroup of cirrhotic patients at increased risk of advanced renal injury during hospitalization.

The prognostic relevance of AKI severity in cirrhosis has been consistently demonstrated. Higher AKI stages are associated with significantly increased mortality and worse clinical outcomes compared with mild AKI or no AKI [1,2,15]. Moreover, heterogeneity within KDIGO stages further influences prognosis, underscoring the clinical importance of accurately identifying patients at risk for progression to severe AKI [4]. In this context, factors associated specifically with stage 2–3 AKI warrant careful investigation.

**Figure 2:**
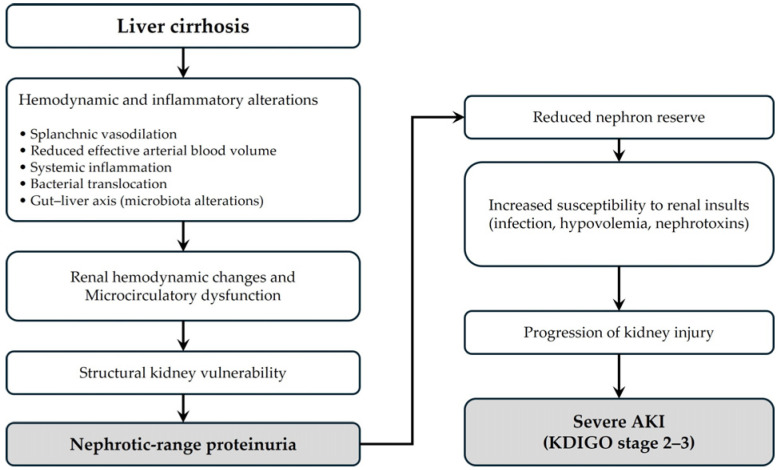
Proposed pathophysiological mechanisms linking nephrotic-range proteinuria and severe acute kidney injury (AKI) in patients with liver cirrhosis. Cirrhosis-related hemodynamic alterations, systemic inflammation, and gut–liver axis dysfunction contribute to renal microcirculatory impairment and structural kidney vulnerability. Nephrotic-range proteinuria reflects underlying glomerular injury and reduced nephron reserve, thereby increasing susceptibility to additional renal insults and progression to severe AKI.

The pathophysiological mechanisms underlying our findings are multifactorial. Cirrhosis-related AKI has traditionally been viewed as predominantly functional, driven by circulatory dysfunction and systemic vasodilation [6,7,8]. However, contemporary consensus statements emphasize that AKI in cirrhosis is heterogeneous and may include structural renal injury beyond classic HRS [9]. Nephrotic-range proteinuria reflects significant glomerular damage and may indicate reduced nephron reserve. Proteinuria itself has been implicated in tubular inflammation, oxidative stress, and progressive renal injury [11]. Thus, patients with substantial proteinuria may be more vulnerable to additional renal insults such as infection, hemodynamic instability, or nephrotoxic exposure, facilitating progression from mild creatinine elevation to more advanced AKI stages. To facilitate conceptual understanding, we provide a schematic illustration of the proposed pathophysiological framework linking nephrotic-range proteinuria and severe AKI in cirrhosis (Figure 2). This figure integrates hemodynamic alterations, systemic inflammation, and gut–liver axis dysfunction into a unified model of renal vulnerability and disease progression. Beyond structural glomerular injury, emerging evidence suggests that systemic inflammation and the gut–liver–kidney axis play important roles in the development of AKI in cirrhosis [16,17]. Increased intestinal permeability and bacterial translocation, as well as alterations in gut microbiota composition, may trigger systemic inflammatory responses, leading to renal microcirculatory dysfunction and structural kidney injury. In this context, nephrotic-range proteinuria may reflect not only intrinsic glomerular damage but also a state of heightened susceptibility to inflammatory and hemodynamic stress, which may predispose patients to progression to severe AKI.

Traditionally, AKI in cirrhosis has been conceptualized primarily as a functional disorder driven by circulatory dysfunction, exemplified by hepatorenal syndrome. However, emerging evidence suggests that AKI in cirrhosis is heterogeneous and may involve varying degrees of structural kidney injury. In this context, nephrotic-range proteinuria may serve as a marker of underlying glomerular damage rather than merely reflecting hypoalbuminemia or hemodynamic alterations. Therefore, proteinuria may capture a dimension of renal vulnerability that is not fully explained by circulatory dysfunction alone. From this perspective, nephrotic-range proteinuria should not be viewed simply as a laboratory abnormality but rather as an indicator of structural renal susceptibility in patients with cirrhosis.

Although serum albumin levels were lower in the nephrotic-range proteinuria group at baseline, serum albumin was not included separately in the multivariable model because it is a component of the Child–Pugh classification. Nevertheless, the observed association between nephrotic-range proteinuria and severe AKI suggests that the prognostic relevance of proteinuria may not be explained solely by hypoalbuminemia or oncotic pressure alterations. Rather, proteinuria may serve as a more direct marker of structural renal vulnerability than serum albumin concentration, which is influenced by both hepatic synthetic function and systemic inflammation.

Consistent with prior literature, CKD and advanced liver dysfunction were associated with severe AKI in our study [1,15]. Although CKD was adjusted for in the multivariable model, residual confounding related to underlying kidney disease severity cannot be fully excluded, as nephrotic-range proteinuria may reflect more advanced or active renal pathology not fully captured by the definition of CKD. The bidirectional relationship between CKD and AKI has been well established, with reduced renal reserve amplifying susceptibility to acute injury and adverse outcomes [10]. Importantly, the association between nephrotic-range proteinuria and severe AKI persisted even after adjustment for CKD and Child–Pugh class, suggesting that proteinuria may provide incremental prognostic information beyond traditional markers of renal and hepatic severity, although residual confounding cannot be fully excluded. ICU admission was also associated with severe AKI, likely reflecting the contribution of critical illness and hemodynamic instability. However, ICU admission may act as both a marker of illness severity and a consequence of clinical deterioration, which complicates causal interpretation.

From a clinical perspective, our findings suggest that routine quantitative assessment of proteinuria may help identify cirrhotic patients at heightened risk for severe renal deterioration. In particular, nephrotic-range proteinuria could be incorporated as a simple and readily available marker in risk stratification frameworks to identify high-risk patients at the time of hospital admission. Such patients may benefit from closer hemodynamic monitoring, early avoidance of nephrotoxic exposures, and more aggressive management of precipitating factors such as infection or volume depletion. Furthermore, proteinuria may complement existing clinical risk indicators, such as liver disease severity and baseline kidney function, and may be integrated into future risk prediction models to improve early risk assessment. However, prospective validation studies are required before proteinuria can be integrated into formal risk prediction models.

### Limitations

Several limitations merit consideration. First, this was a single-center retrospective study, which may limit generalizability and precludes causal inference. Second, quantitative proteinuria testing was not systematically performed in all hospitalized patients with cirrhosis. Therefore, inclusion in the study may have been influenced by clinical suspicion of renal dysfunction or disease severity, introducing potential selection bias. As a result, the study population may represent a clinically selected subgroup rather than the entire population of patients with cirrhosis, which may limit generalizability and potentially overestimate the observed association. Third, the definition of baseline serum creatinine may have introduced misclassification of kidney function and AKI staging. Although we used a hierarchical approach based on pre-admission values to approximate stable kidney function, alternative definitions could yield different results. The absence of sensitivity analyses using alternative baseline definitions may affect the robustness of the findings. Future studies incorporating sensitivity analyses with varying baseline creatinine definitions are warranted. Fourth, residual confounding cannot be excluded despite multivariable adjustment, as detailed information on vasoconstrictor therapy, fluid management strategies, and nephrotoxic exposures was not uniformly available. In addition, although CKD was included in the adjustment, nephrotic-range proteinuria may reflect more advanced or active renal pathology not fully captured by the definition of CKD. Fifth, because UPCR measurements included values obtained at admission, early kidney injury may have influenced proteinuria levels, introducing potential reverse causation. This temporal ambiguity limits the ability to establish a clear exposure–outcome sequence and may bias the observed association. Sixth, we were unable to reliably distinguish between different AKI subtypes, such as hepatorenal syndrome-associated AKI, prerenal AKI, or intrinsic renal injury. Therefore, the observed associations may vary across underlying AKI mechanisms, which limits mechanistic interpretation and clinical specificity. Seventh, nephrotic-range proteinuria was defined based on a UPCR ≥3.5 g/g without systematic confirmation by 24-h urine collection or lipid profiles; thus, our findings reflect the presence of nephrotic-range proteinuria rather than the full clinical phenotype of nephrotic syndrome. In addition, urine output criteria were not incorporated into AKI staging due to incomplete documentation. Eighth, given the limited number of events in relation to the number of covariates, the multivariable model may be prone to overfitting despite acceptable diagnostic indicators. Although model diagnostics indicated acceptable calibration and no significant multicollinearity, the stability of the estimates may be limited and the effect estimates may be somewhat optimistic. Therefore, the results should be interpreted with caution, and future studies with larger sample sizes and external validation are needed to confirm these findings. Ninth, subgroup analyses according to CKD status or liver disease severity were not performed due to limited sample size and event numbers, which may compromise model stability. As a result, potential heterogeneity of the association across clinically relevant subgroups could not be evaluated. Future studies with larger cohorts are needed to determine whether the observed associations differ across these subgroups. Finally, the observational design prevents determination of whether proteinuria is a modifiable risk factor or a marker of pre-existing renal susceptibility.

Despite these limitations, this study has notable strengths. We applied standardized KDIGO criteria for AKI definition [14], incorporated contemporary consensus perspectives in cirrhosis-related AKI [8,9], and adjusted for multiple clinically relevant confounders. Moreover, by focusing specifically on severe AKI (stage 2–3) rather than overall AKI occurrence, we addressed the phenotype most strongly associated with adverse prognosis [1,2]. Together, these findings support the hypothesis that structural renal vulnerability may be involved in advanced AKI phenotypes in cirrhosis.

## 5. Conclusions

In hospitalized patients with liver cirrhosis, nephrotic-range proteinuria was associated with an increased likelihood of developing severe AKI. Beyond a statistical association, proteinuria may serve as a clinically accessible marker of underlying structural renal vulnerability, identifying patients at increased risk of progression to advanced kidney injury. From a clinical perspective, early identification of high-risk patients through routine quantitative proteinuria assessment may support more proactive monitoring and timely intervention. Although causality cannot be inferred from this observational study, our findings provide a clinically meaningful basis for incorporating proteinuria into risk stratification frameworks in cirrhosis. Further prospective, multicenter studies are warranted to validate these findings and to clarify the role of proteinuria in guiding clinical decision-making.

## Figures and Tables

**Table 1:** Baseline Characteristics According to the Presence of Nephrotic-range Proteinuria.

Variable	Without Nephrotic-Range Proteinuria (*n* = 352)	With Nephrotic-Range Proteinuria (*n* = 56)	SMD	*p*-Value
Age (years)	64.48 ± 10.22	65.36 ± 10.99	0.08	0.576
Sex (Male)	236 (67.0)	42 (75.0)	0.18	0.302
Diabetes mellitus	136 (38.6)	23 (41.1)	0.05	0.842
Hypertension	160 (45.5)	26 (46.4)	0.02	1.000
Chronic kidney disease	108 (30.7)	31 (55.4)	0.50	<0.001
Sepsis	123 (34.9)	24 (42.9)	0.16	0.319
ICU admission	125 (35.5)	17 (30.4)	−0.11	0.548
Ascites	97 (27.6)	16 (28.6)	0.02	1.000
Variceal bleeding	60 (17.0)	11 (19.6)	0.07	0.775
Total bilirubin (mg/dL)	2.78 ± 1.58	2.74 ± 2.00	−0.02	0.607
Albumin (g/dL)	3.02 ± 0.50	2.79 ± 0.54	−0.44	0.004
INR	1.46 ± 0.24	1.48 ± 0.28	0.09	0.750
Child-Pugh class (A)	125 (35.5)	22 (39.3)	0.18	0.458
Child-Pugh class (B)	156 (44.3)	20 (35.7)		
Child-Pugh class (C)	71 (20.2)	14 (25.0)		
Baseline creatinine (mg/dL)	1.03 ± 0.34	1.20 ± 0.39	0.45	0.003
UPCR (g/g)	1.62 ± 0.89	5.10 ± 1.35	3.05	<0.001
Length of stay (days)	9.72 ± 6.90	10.64 ± 6.85	0.13	0.222

Values are presented as mean ± standard deviation or frequency (percent). Baseline creatinine was defined as the lowest serum creatinine value measured within 7 days prior to admission; if unavailable, the lowest stable value documented within the preceding 3 months was used. ICU, intensive care unit; INR, international normalized ratio; SMD, standardized mean difference; UPCR, urine protein-to-creatinine ratio.

**Table 2:** AKI Severity According to the Presence of Nephrotic-range Proteinuria.

AKI Stage	Without Nephrotic-Range Proteinuria (*n* = 352)	With Nephrotic-Range Proteinuria (*n* = 56)	*p*-Value
Stage 0	129 (36.6)	10 (17.9)	0.004
Stage 1	146 (41.5)	24 (42.9)	
Stage 2	53 (15.1)	12 (21.4)	
Stage 3	24 (6.8)	10 (17.9)	
Severe AKI (Stage 2–3)	77 (21.9)	22 (39.3)	0.008

AKI was defined according to KDIGO criteria based on serum creatinine changes. Severe AKI was defined as KDIGO stage 2–3. AKI, acute kidney injury.

**Table 3:** Multivariable Logistic Regression Analysis for Severe AKI.

Variable	β	Adjusted OR (95% CI)	*p*-Value
Nephrotic-range proteinuria	0.820	2.27 (1.17–4.41)	0.015
Age (per year)	−0.007	0.99 (0.97–1.02)	0.559
Sex (male)	−0.208	0.81 (0.48–1.37)	0.437
Diabetes mellitus	−0.269	0.76 (0.45–1.29)	0.313
Hypertension	0.057	1.06 (0.65–1.73)	0.818
Chronic kidney disease	0.813	2.26 (1.33–3.81)	0.002
Sepsis	0.397	1.49 (0.91–2.45)	0.118
ICU admission	0.708	2.03 (1.22–3.39)	0.007
Child–Pugh class B (vs A)	0.403	1.50 (0.74–3.02)	0.262
Child–Pugh class C (vs A)	1.253	3.50 (1.37–8.93)	0.009

Multivariable logistic regression was performed with severe AKI (KDIGO stage 2–3) as the dependent variable. Child–Pugh class A was used as the reference category. CI, confidence interval; ICU, intensive care unit.

## Data Availability

The data presented in this study are available on request from the corresponding author under ethical and legal restrictions related to patient confidentiality.

## Supplementary Materials

The following supporting information can be downloaded at: https://www.mdpi.com/article/10.3390/medicina62040797/s1, Figure S1. Study flow diagram illustrating patient selection and reasons for exclusion. UPCR, urine protein-to-creatinine ratio.

## Abbreviations

The following abbreviations are used in this manuscript:

AKI	Acute Kidney Injury
CKD	Chronic Kidney Disease
CI	Confidence Interval
EMR	Electronic Medical Records
HRS	Hepatorenal Syndrome
ICU	Intensive Care Unit
INR	International Normalized Ratio
KDIGO	Kidney Disease: Improving Global Outcomes
LC	Liver Cirrhosis
OR	Odds Ratio
SMD	Standardized Mean Difference
UPCR	Urine Protein-to-Creatinine Ratio
