# Supplementary material for: Risk of Severe Acute Kidney Injury According to the Presence of Nephrotic-Range Proteinuria in Patients with Liver Cirrhosis: A Retrospective Cohort Study (2016–2025)
Source: Medicina (Kaunas). 2026 Apr 21;62(4):797. doi: 10.3390/medicina62040797 (PMC13117935; doi:10.3390/medicina62040797)
Supplement: Supplementary file 1 [file medicina-62-00797-s001.zip › medicina-4251302-supplementary.pdf]

Supplementary Material

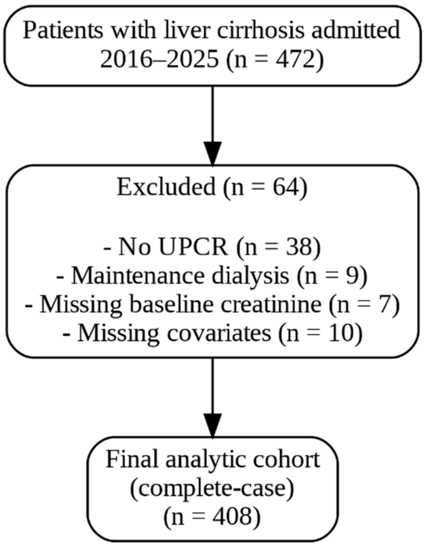

Figure S1. Study flow diagram illustrating patient selection and reasons for exclusion. UPCR, urine protein-to-creatinine ratio.
